# The Light Wavelength, Intensity, and Biasing Voltage Dependency of the Dark and Photocurrent Densities of a Solution-Processed P3HT:PC_61_BM Photodetector for Sensing Applications

**DOI:** 10.3390/nano14181496

**Published:** 2024-09-14

**Authors:** Farjana Akter Jhuma, Kentaro Harada, Muhamad Affiq Bin Misran, Hin-Wai Mo, Hiroshi Fujimoto, Reiji Hattori

**Affiliations:** 1 Major of Device Science and Engineering, Interdisciplinary Graduate School of Engineering Sciences, Kyushu University, Fukuoka 8190395, Japan; jhuma.farjana.akter.233@s.kyushu-u.ac.jp (F.A.J.);; 2 OPERA Solutions Inc., 5-5 Kyudai-shimmachi, Nishi-ku, Fukuoka 8190388, Japan

**Keywords:** organic photodetector, LED, photocurrent density, dark current density, on/off ratio

## Abstract

The promising possibility of an organic photodetector (OPD) is emerging in the field of sensing applications for its tunable absorption range, flexibility, and large-scale fabrication abilities. In this work, we fabricated a bulk heterojunction OPD with a device structure of glass/ITO/PEDOT:PSS/P3HT:PC_61_BM/Al using the spin-coating process and characterized the dark and photocurrent densities at different applied bias conditions for red, green, and blue incident LEDs. The OPD photocurrent density exhibited a magnitude up to 2.5–3 orders higher compared to the dark current density at a −1 V bias while it increased by up to 3–4 orders at zero bias conditions for red, green, and blue lights, showing an increasing trend when a higher voltage is applied in the negative direction. Different OPD inner periphery shapes, the OPD to LED distance, and OPD area were also considered to bring the variation in the OPD dark and photocurrent densities, which can affect the on/off ratio of the OPD–LED hybrid system and is a critical phenomenon for any sensing application.

## 1. Introduction

Photodetectors are sensing devices that convert incident light to corresponding electrical signals. Organic-based photodetectors (OPDs) have attracted researchers’ attention immensely during the past decade for their enormous potential due to their advantages, such as material tunability, low-cost solution processability, mechanical flexibility, stretchability, lightweight, and applicability to large-area displays. These advantages have made OPDs versatile in combination with conventional photodetectors with inorganic materials [1,2,3,4,5]. The ability of broadband and narrowband detection has allowed OPDs to be used in optical sensing and imaging, health monitoring, artificial vision, and photonic communication [6,7,8,9]. Lee et al. demonstrated an all-day-wearable health monitoring system based on a broadband OPD [10]. High-resolution image sensors with ultra-high flexibility have also been demonstrated using an OPD (Zalar et al.) [11].

Generally, the response spectral range of an OPD depends on photon harvesting in the organic active layer material. OPDs made from the combination of semiconducting polymers (electron donors) and buckminsterfullerene derivatives (electron acceptors) have been proven to be capable of high detectivity and fast temporal response by many researchers [12,13,14]. The bulk heterojunction of an OPD, prepared from the electron donor and acceptor composite, undergoes several processes like photon absorption, the generation of charge-transfer (CT) excitons, and the dissociation of excitons before transporting and collecting the charge carriers under light illumination. The magnitude of the photocurrent density (*J_ph_*) induced by these processes depends highly on the intensity of the incident light [15]. Although the bulk heterojunction OPD suffers from a low response speed and high dark current issues, the photocurrent can be increased by minimizing the recombination loss [16]. For any kind of sensing application, the OPD must possess the combination of a low dark current and a high photocurrent along with a high on/off ratio to achieve an excellent photo response in reverse bias conditions. 

In this paper, we demonstrated the fabrication and realization of typical poly(3-hexylthiophene-2,5-diyl)) (P3HT): [6,6]-phenyl C_61_-butyric acid methylester (PC_61_BM) bulk heterojunction-based OPDs using the easy and cost-effective solution process. The combination of a P3HT donor and PC61BM acceptor supports efficient charge carrier generation and transfer properties along with photoresponses in the visible region, enabling its useful application as a photodetector [13,17]. To improve the sensitivity of the photodetector, it is very necessary to carefully consider the dark and photocurrent densities of the detector itself, which highly depend on the illumination conditions, i.e., the light wavelength and intensity and the OPD’s operating conditions. To understand these effects, the dark and photocurrent densities of the OPD at different illumination conditions were characterized in this work. In addition, the effects of various designs and areas of the OPD were also considered to optimize the device’s structure, which is crucial for future use in sensing applications. 

## 2. Device Fabrication and Characterization

Commercially available indium tin oxide (ITO)-coated glass substrates with an ITO thickness of 100 nm were precleaned and subjected to a photolithography process, followed by wet etching for the patterning of the transparent ITO electrode. The patterned ITO substrates were then cleaned in an ultrasonic bath of DI water, detergent solution, acetone, and isopropyl alcohol (IPA) several times for 5 min each sequentially. The cleaned substrates were then subjected to UV/ozone treatment for 20 min to remove any remaining organic residues from the substrates. As the hole transport layer, the filtered poly(3,4-ethylenedioxythiophene)-poly(styrene sulfonate) (PEDOT:PSS) (Clevious P VP AI 4083, Ossila Limited, Sheffield, UK) solution was spin-coated on the patterned ITO substrate at 3000 rpm for 60 s (thickness ~80 nm) and baked at 200 °C for 10 min in the air. The P3HT (FUJIFILM Wako Chemicals, Osaka, Japan) and PC_61_BM (Sigma Aldrich, St. Louis, MO, USA) blend was used as the bulk heterojunction active layer of the OPD. Both materials were dissolved in chlorobenzene in a 1.8:1 ratio, and the prepared solution was kept on magnetic stirring for 2 days before deposition. The P3HT:PC_61_BM solution was deposited over PEDOT:PSS in a spin-coating process at a speed of 1000 rpm for 50 s, followed by baking at 80 °C for 5 min in a N_2_ environment. Subsequently, a 100 nm Al cathode was deposited over the active layer (thickness ~130 nm) through a metal mask in the vacuum thermal evaporation process and then annealed at 150 °C for 5 min in a N_2_ environment. Finally, the fabricated device was encapsulated with a 100 nm silicon nitride (SiN) thin film layer by plasma-enhanced chemical vapor deposition and subjected to the electrical measurement procedure. 

**Figure 1:**
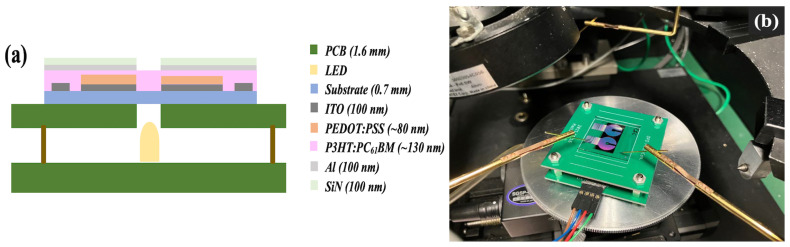
(**a**) Schematic of *J–V* measurement setup for OPD. (**b**) Actual image of parameter analyzer and micro-probing system.

The dark and photocurrent measurements of the OPD were performed with a precision semiconductor parameter analyzer (Agilent 4156C, Agilent Technologies, Santa Clara, CA, USA) and a micro probing system (K157MP, Kyowa Riken, Tokyo, Japan) at room temperature. A commercially available RGB LED (HV-5RGB25, Inolux, Santa Clara, CA, USA) was used as the light source to illuminate the OPD while performing the measurement. The OPD was illuminated with three different wavelengths, namely red (λ = 624 nm; luminous intensity = 150.06 mcd), green (λ = 525 nm; luminous intensity = 7.84), and blue (λ = 470 nm; luminous intensity = 1.57 cd), for the photocurrent measurement with supply current controlled irradiation intensity. The luminous intensities of red, green, and blue LEDs were calculated from the illuminance measured using the light meter (AS ONE LM-332) and the distance between the LED and the light meter. The schematic for the measurement system and the actual measurement setup are shown in Figure 1. 

## 3. Results and Discussions

**Figure 2:**
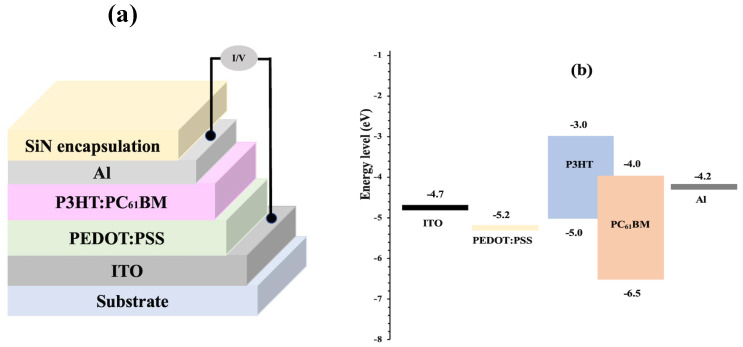
(**a**) Schematic of OPD device structure; (**b**) energy level diagram of OPD layers.

The layer stacks of the device, the chemical structures of the donor and acceptor materials, and the energy diagram of the OPD layer materials are shown in Figure 2. The hole transport layer PEDOT:PSS can support efficient carrier injection and charge collection at the electrode terminal [18]. A typical P3HT:PC_61_BM blend was adopted for the active layer because it has been extensively used for organic photovoltaic devices even though the power conversion efficiency is limited by a biomolecular recombination loss [19]. Followed by an aluminum top contact, the SiN encapsulation layer was used to protect the device from environmental degradation.

### 3.1. Effect of LED Wavelength on OPD J–V Characteristics

**Figure 3:**
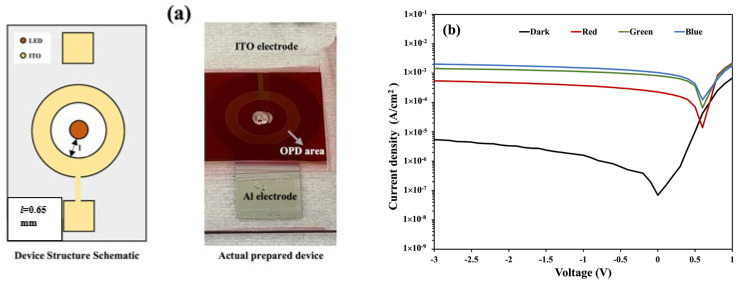
(**a**) A schematic of the structure and actual image of the fabricated D1 device. (**b**) The *J–V* characteristics of the D1 device.

Figure 3 shows the OPD device (D1) structure schematic, the image of the actual device, and the current density–voltage (*J–V*) characteristics both in dark and illuminated conditions. The device was illuminated with green, red, and blue light from the rear side, as shown in Figure 1. The three LEDs have wavelengths of 470 nm (*λ_blue_*), 525 nm (*λ_green_*), and 632 nm (*λ_red_*), where *λ_blue_* < *λ_green_* < *λ_red_*. The photocurrent densities in the OPD are affected by the illumination of different LED colors, which can be observed in Figure 3.

The dark current density depends, e.g., on the degree of minority carrier injections such as the electron injection from the PEDOT:PSS layer to the lowest unoccupied molecular orbital (LUMO) of P3HT or PC_61_BM and the hole injection from the Al cathode to the highest occupied molecular orbital (HOMO) of the P3HT or PC_61_BM [16]. The dark current density and photocurrent density for different LED colors at different reverse bias voltages are summarized in Table 1. Under illumination, the OPD photocurrent density shows up to 3–4 orders of magnitude higher than the dark current density at a zero bias condition, whereas it is only 2.5–3 orders of magnitude higher than the dark current at a −1 V reverse bias for red, green, and blue incident lights.

**Figure 4:**
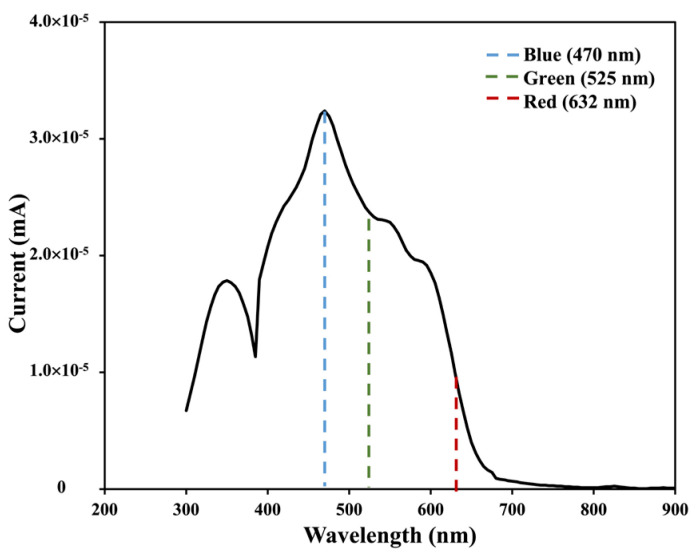
Photocurrent versus incident wavelength for prepared P3HT:PC_61_BM OPD device.

In many studies, it was found that for the P3HT:PC_61_BM heterostructure, the electron donor P3HT has large absorption in a visible wavelength region (380–660 nm), whereas the electron acceptor PC_61_BM absorbs light in a UV wavelength region (280–380 nm), and their blended structure results in an absorption peak around 470–480 nm [20,21,22]. Due to the shoulder of the characteristic absorption bands, photons incident on the OPD from an LED with a wavelength of 470 nm (*λ_blue_*) can efficiently generate excitons both in P3HT and PC_61_BM, whereas photons incident from green and red LEDs (*λ_green_* and *λ_red_*, respectively) can mainly produce excitons in the P3HT acceptor only. The illumination of shorter wavelengths (*λ_blue_*) can thus contribute to a larger number of CT exciton generations, causing a higher photocurrent density compared to that of longer wavelengths (*λ_green_* and *λ_red_*). The relation between the photocurrent wavelength versus incident wavelength for the prepared P3HT:PC_61_BM heterostructure in Figure 4 shows a higher current value near 470 nm corresponding to *λ_blue_*, and it degrades gradually for longer wavelengths corresponding to *λ_green_* and *λ_red_.*

### 3.2. Effect of LED Intensity and OPD Biasing 

**Figure 5:**
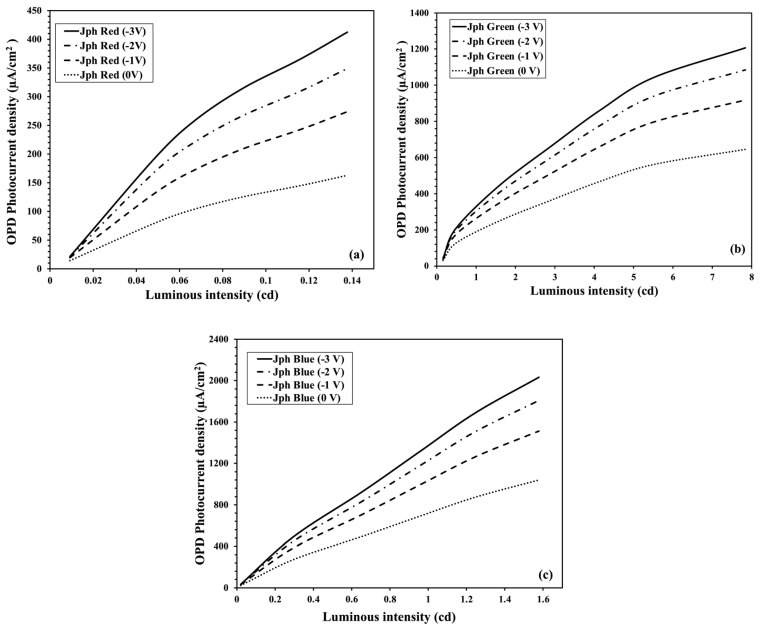
OPD photocurrent density vs. LED luminous intensity curves with applied reverse bias condition for (**a**) red LED, (**b**) green LED, and (**c**) blue LED.

For any sensing application, the OPD needs to exhibit a stable and persistent performance over a wide range of light intensity. In this section, we discuss the performance of the OPD for increasing the LED’s luminous intensity under different biasing conditions. The OPD photocurrent density, *J_ph_*, highly depends on the intensity of the incident LED illumination. The magnitude of *J_ph_* for a particular excitation wavelength, *λ*, and the light intensity, *I_L_*, can be described by

(1) $$\left|J_{ph}\right|=EQE\times \frac{q\lambda }{hc}\;I_{L},$$

where *EQE* is the external quantum efficiency, *h* is the Planck constant, *c* is the light speed, and *q* is the elementary charge. The *EQE* depends on the absorption of photons, generation of excitons to be converted into free charge carriers, and collection of carriers before recombination. Equation (1) indicates a proportional dependence of the photocurrent density on the light intensity as far as the *EQE* is unchanged. With increasing light intensity, the number of incident photons and thus the number of photogenerated carriers increases, which, in turn, increases the *J_ph_* [23,24]. Figure 5 shows the variation in our OPD photocurrent densities, *J_ph_* (red), *J_ph_* (green), and *J_ph_* (blue), with the LED wavelengths *λ_red_*, *λ_green_,* and *λ_blue_*, respectively, for different OPD biasing voltages with increasing LED luminous intensities, *I_L_*. As a trend, the OPD photocurrent densities are roughly linearly correlated with the incident light intensities. However, nonlinearity is observed as the light intensities are widely varied. A possible explanation of the deviation from the linearity is a decrease in the *EQE* due to the biomolecular recombination losses for an increasing charge carrier density [16,19]. Another reason for the deviation is that our LED is a point light source, so it is not perpendicularly incident on the OPD surface, while the luminous intensities in Figure 5 were calculated assuming a planer light source, i.e., the photon numbers incident on the OPD are assumed to be uniform over the area. We will discuss the impact of the light path in Section 3.3.

A reverse bias voltage in general enhances the strength of the electric field at the junction of photodetector devices as it expedites the carriers’ drift velocity and reduces the carriers’ transit time, enhancing the charge collection and lowering the probability of carrier recombination losses [25]. The transit time, which is inversely proportional to the electric field, can be defined by

(2) $$\tau_{t\;}=\frac{l^{2}}{\mu V},\;$$

where *V* is the bias voltage, *l* is the space charge layer length at the junction of a conventional photodetector, and *μ* is the carrier mobility [26]. Although the conventional semiconductor theory is not fully applicable to organic bulk heterojunction devices, the effect of an external field on the carrier collection and the reduction in biomolecular recombination is analogous. Moreover, the dissociation of CT excitons coupled with a coulombic force requires an external field [27]. Due to the combined effects of field dependency, the magnitude of the OPD photocurrent *I_p_* increases with the reverse bias applied, which can be observed in Figure 5, where the OPD photocurrent magnitude increases for a higher reverse bias (−1 V to −3 V) for an LED with red, green, and blue colors.

### 3.3. Effect of OPD Design

In this section, we discuss the OPD’s performance for different design variations. In sensing applications, the design of the OPD plays an important role for the sensors to perform efficiently. The performance of the sensor is significantly influenced by the design parameters of the OPD, including the shape of the OPD’s surface near the LED, the distance between the LED and the OPD, and the OPD’s surface area. These parameters can critically affect the OPD’s dark and photocurrent densities as well as the on/off ratio (ratio of photocurrent density to dark current density), which are necessary for optimizing the detector’s performance in any sensing applications. Variations in the OPD’s design can introduce noise, potentially affecting the signal acquisition procedure. Therefore, careful optimization of these parameters is very much needed to ensure reliable signal detection and overall sensor efficiency.

Table 2 shows the design configuration summary of each device used in this study.

#### 3.3.1. OPD Inner Periphery Shape

**Figure 6:**
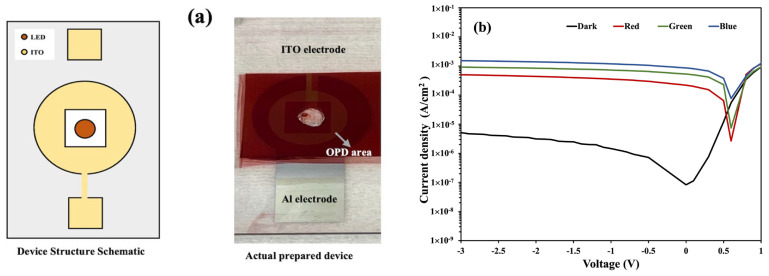

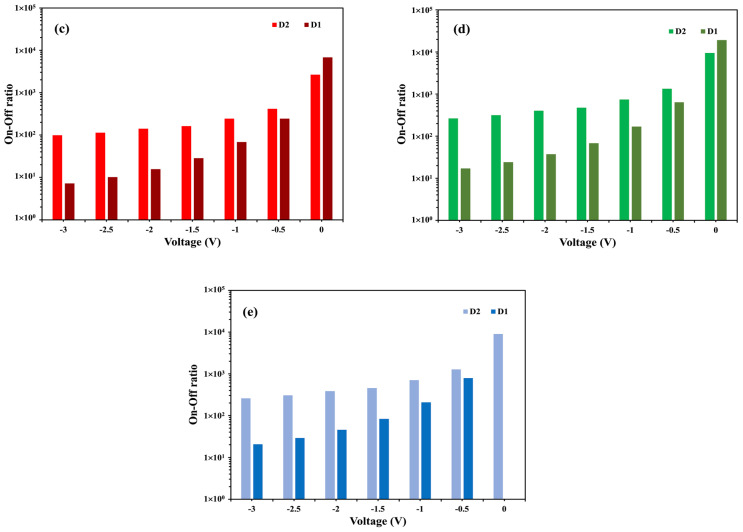
(**a**) A schematic of the structure and the image of the fabricated D2 device. (**b**) The *J–V* characteristics of the D2 device. (**c**–**e**) A comparison of the on/off ratio between the D1 and D2 devices for red, green, and blue LEDs, respectively.

Khan et al. showed different sensor geometries (OPD shapes) used for photoplethysmography (PPG) signal analysis [28]. In this work, we used two different geometries of the inner periphery of the OPD active area, which is closer to the LED. As shown in Figure 3, device D1 has a circular inner periphery OPD area, whereas D2 has a rectangular inner geometry (see Figure 6). Most commercial LEDs used in any system have a rectangular shape rather than a dome shape. In the case of rectangular LEDs, the rectangular inner periphery of the OPD area is convenient as it can collect LED light from all directions at equal distances. In contrast, for the dome-shaped LED, a circular OPD inner geometry is preferable from the same context. The schematic of the structure, image of the fabricated D2 device, the D2 *J–V* characteristics, and graphs of the on/off ratio for both D1 and D2 devices are shown in Figure 6. 

#### 3.3.2. Distance from LED to OPD

**Figure 7:**
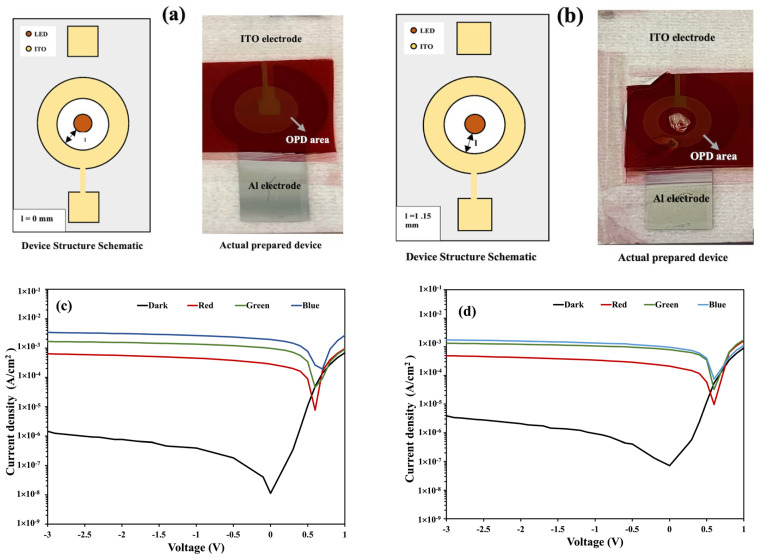
(**a**,**b**) Schematics of the structures and images of the fabricated devices D3 (*l* = 0 mm) and D4 (*l* = 1.15 mm), respectively; (**c**,**d**) the *J–V* characteristics of devices D3 and D4, respectively.

The distance from the LED and the OPD is also an important factor to consider. Affiq et al. suggested that the distance from the light source to the detector in a reflectance photoplethysmography (PPG) sensor should be kept at less than 3 mm for efficient signal detection [29]. Here, we investigated three devices by varying the distance from the LED to the OPD. Devices D3, D1, and D4 have distances (*l*) of 0 mm, 0.65 mm, and 1.15 mm, respectively, from the LED surface to the inner periphery of the OPD. The *J–V* curve of device D1 is shown in Figure 3, whereas the schematics of the structures and the *J–V* characteristics of devices D3 and D4 are shown in Figure 7. The dark and photocurrent densities of these three devices at zero bias conditions (0 V) are summarized in Table 3. 

The dark current density increases with the increase in the distance from the OPD to LED, whereas the photocurrent density decreases with an increasing distance. According to the theoretical inverse-square law of radiation, the measured intensity of light is inversely proportional to the source-to-detector distance squared (*l*^2^) [30]. As the distance from the LED to OPD increases, it reduces the number of photons absorbed by the OPD bulk heterojunction materials, while it increases the possibility of noise associated with light scattering, which, in turn, reduces the photocurrent density and increases the dark current density.

**Figure 8:**
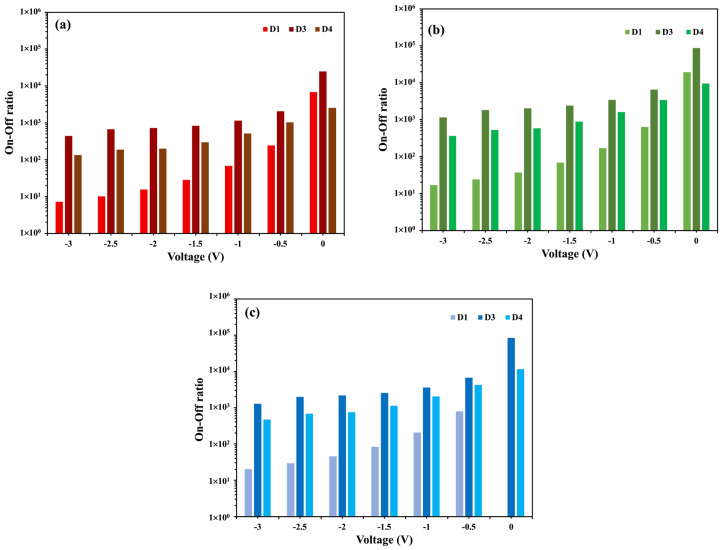
(**a**–**c**) Comparison of on/off ratio between D1 (*l* = 0.65 mm), D3 (*l* = 0 mm), and D4 (*l* = 1.15 mm) for red, green, and blue LEDs, respectively.

The graphs of the on/off ratio comparison between devices D1, D3, and D4 are shown in Figure 8 for red, green, and blue incident illumination. A decreasing trend in the on/off ratio is observed when the source-to-detector distance *(l)* (D3 > D1 > D4) is increased. As summarized in Table 3, the trend in the on/off ratio is the consequence of the reducing photocurrent density and the increasing dark current density with an increasing *l.*

#### 3.3.3. OPD Area

**Figure 9:**
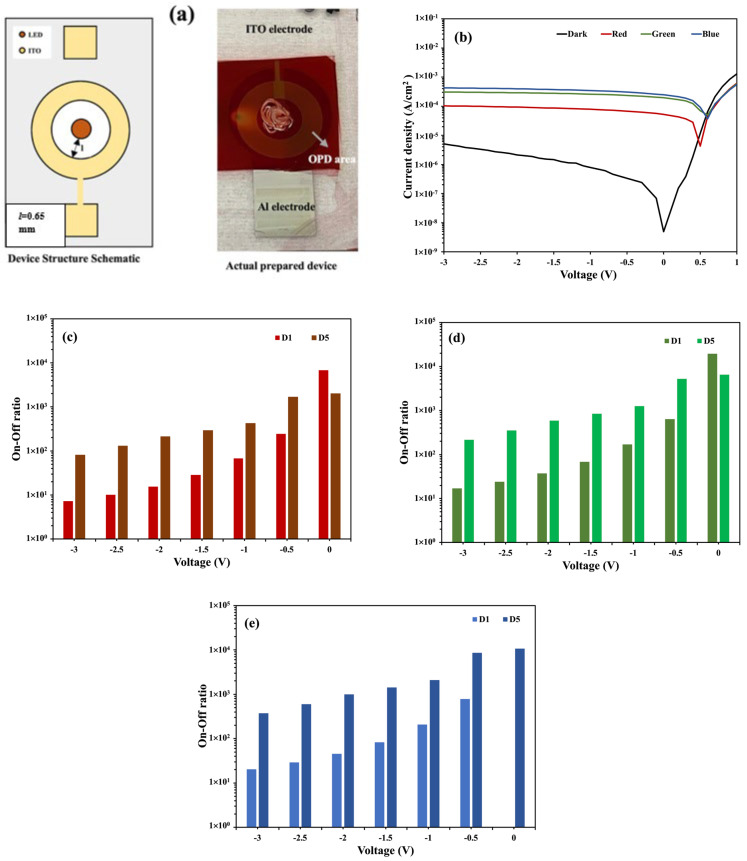
(**a**) A schematic of the structure and images of the fabricated D5 device. (**b**) The *J–V* characteristics of the D5 device. (**c**–**e**) A comparison of the on/off ratio between devices D1 and D5 for red, green, and blue LEDs, respectively.

One of the advantages of an organic photodetector is that it can be fabricated over a large area. The OPD’s area can affect its dark and photocurrent densities. We investigated the effect of the area size by comparing two OPD devices with active areas of 16 mm^2^ (D1) and 32 mm^2^ (D5) with the same inner periphery and distance from the incident LED (*l* = 0.65 mm). The *J–V* curve of device D5 and graphs of the on/off ratio comparison between devices D1 and D5 are shown in Figure 9. 

Increasing the active area allows more photons to be absorbed in the OPD and contributes to a larger number of charge carriers generated in total. If the LED incident is normal to the OPD active area and the light intensity is uniform over the area, the value of the photocurrent density does not depend on the area according to the definition (A/cm^2^). In our OPD-LED hybrid system, the LED is a point source, i.e., the light spreads radially and it follows a path through reflection to reach the OPD active area (see Figure 1). Therefore, a larger OPD area has some disadvantages in uniform light illumination. In addition, it is known that an increase in the area can enhance the probability of defects that accelerate electron and hole recombination at a low built-in junction potential, and thus, the photocurrent density in organic bulk heterojunction is often dependent on the area size [31]. The above two factors should be the reasons behind the lower photocurrent density of device D5, as seen in Figure 9b, compared to that of the other devices. The inferior zero bias on/off ratio of D5 compared to that of D1 seen in Figure 9c–e can be attributed to the lower photocurrent density in D5. However, as we discussed earlier, applying a reverse bias voltage decreases the probability of carrier recombination losses. This explains the superior on/off ratio of the D5 device at reverse bias conditions. However, at the highest reverse bias (−3 V), the on/off ratio of D5 is lowered again due to the increasing dark current possibly associated with minority carrier injections. 

## 4. Conclusions

In this work, we demonstrated the performance of an OPD-LED hybrid system, i.e., a combination of an OPD and an incident LED. The role of the LED forward current and different OPD biasing conditions was studied. The results indicate a higher magnitude of OPD photocurrent at higher reverse bias conditions due to enhanced charge carrier generation and collection. The OPD *J–V* characteristics were investigated for different LEDs with different wavelengths, and a higher current density was observed for a shorter incident wavelength, i.e., for the blue LED. We further investigated the OPD *J–V* characteristics and the on/off ratio for different shapes of the OPD inner periphery, different distances between the OPD and the incident LED, and different sizes of the OPD area. The obtained results suggest that the distance between the LED and OPD should be minimized to obtain a low dark current and a high photocurrent, i.e., a high on/off ratio suitable for any kind of sensing application. The variation in the OPD area suggests that the OPD area should be larger if the OPD is used at a zero bias condition, whereas the OPD should possess a smaller area if it is used at a reverse bias condition. The observations presented in this paper can be of great importance for optimizing the OPD structure and OPD operating conditions for different sensing applications.

## Figures and Tables

**Table 1:** Current density of D1 device at different illumination conditions and bias voltages.

Illumination Condition	Current Density (A/cm^2^) at Different Bias Voltages (V)				
	−3 V	−2 V	−1 V	0 V	1 V
Dark	5.43 × 10^−6^	3.35 × 10^−6^	1.60 × 10^−6^	6.93 × 10^−8^	6.78 × 10^−4^
Red	5.46 × 10^−4^	4.70 × 10^−4^	3.75 × 10^−4^	2.27 × 10^−4^	2.19 × 10^−3^
Green	1.43 × 10^−3^	1.31 × 10^−3^	1.13 × 10^−3^	8.17 × 10^−4^	2.06 × 10^−3^
Blue	2.03 × 10^−3^	1.81 × 10^−3^	1.51 × 10^−3^	1.04 × 10^−3^	1.80 × 10^−3^

**Table 2:** Device configuration summary.

Device Label	Area (mm^2^)	Inner Periphery Geometry	LED to OPD Inner Periphery Distance, (*l*) (mm)
D1	16	Round	0.65
D2	16	Rectangular	0.65
D3	16	No inner shape	0
D4	16	Round	1.15
D5	32	Round	0.65

**Table 3:** Current density of OPD device for varying distances from the LED to OPD at different illumination conditions.

Distance from LED to OPD, (*l*) (mm)	Current Density (A/cm^2^) for Different Illumination Conditions			
	Dark	Red	Blue	Green
0.00 (device D3)	1.12 × 10^−8^	2.81 × 10^−4^	1.95 × 10^−3^	9.75 × 10^−4^
0.65 (device D1)	6.93 × 10^−8^	2.27 × 10^−4^	1.04 × 10^−3^	8.17 × 10^−4^
1.15 (device D4)	7.13 × 10^−8^	2.12 × 10^−4^	9.75 × 10^−4^	7.95 × 10^−4^

## Data Availability

The data are contained within this article.
